# Experimental and Numerical Investigation of the Mechanical Properties of 3D-Printed Hybrid and Non-Hybrid Composites

**DOI:** 10.3390/polym15051164

**Published:** 2023-02-25

**Authors:** Tim Heitkamp, Simon Girnth, Sebastian Kuschmitz, Nils Waldt, Günter Klawitter, Thomas Vietor

**Affiliations:** 1 Faculty II, Hochschule Hannover, University of Applied Sciences and Arts, 30459 Hannover, Germany; 2 Institute for Engineering Design, Technische Universität Braunschweig, 38108 Braunschweig, Germany

**Keywords:** continuous fiber, fiber-reinforced additive manufacturing, hybrid composites, finite element analysis (FEA), material extrusion, hybrid fiber-reinforced polymers

## Abstract

Recent research efforts have highlighted the potential of hybrid composites in the context of additive manufacturing. The use of hybrid composites can lead to an enhanced adaptability of the mechanical properties to the specific loading case. Furthermore, the hybridization of multiple fiber materials can result in positive hybrid effects such as increased stiffness or strength. In contrast to the literature, where only the interply and intrayarn approach has been experimentally validated, this study presents a new intraply approach, which is experimentally and numerically investigated. Three different types of tensile specimens were tested. The non-hybrid tensile specimens were reinforced with contour-based fiber strands of carbon and glass. In addition, hybrid tensile specimens were manufactured using an intraply approach with alternating carbon and glass fiber strands in a layer plane. In addition to experimental testing, a finite element model was developed to better understand the failure modes of the hybrid and non-hybrid specimens. The failure was estimated using the Hashin and Tsai–Wu failure criteria. The specimens showed similar strengths but greatly different stiffnesses based on the experimental results. The hybrid specimens demonstrated a significant positive hybrid effect in terms of stiffness. Using FEA, the failure load and fracture locations of the specimens were determined with good accuracy. Microstructural investigations of the fracture surfaces showed notable evidence of delamination between the different fiber strands of the hybrid specimens. In addition to delamination, strong debonding was particularly evident in all specimen types.

## 1. Introduction

Continuous fiber-reinforced composites are becoming increasingly important, especially for structural components in lightweight applications. They are characterized by good strength and stiffness properties combined with a low weight, high corrosion, and fatigue resistance. Typical fields of application for composites include aerospace, automotive, sports, and leisure [1]. 

### 1.1. Continuous Fiber-Reinforced MEX

**Figure 1:**
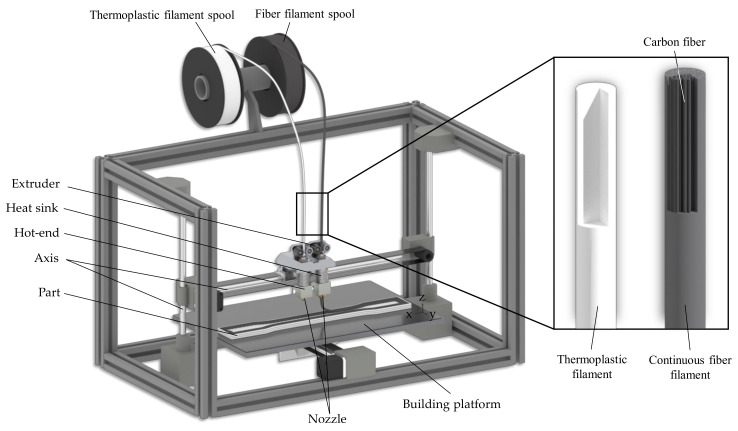
Schematic process principle of continuous fiber-reinforced material extrusion.

The reinforcement of polymeric components using continuous fibers has also been extensively used in additive manufacturing (AM) for several years [2,3]. In this regard, the most prominent approach in the literature is an adapted manufacturing process based on material extrusion (MEX, also Fused Filament Fabrication: FFF, or Fused Deposition Modeling: FDM). MEX is a tool-free additive manufacturing process in which the thermoplastic component is built up layer by layer. The starting material consists of a polymer strand, which is melted in the print head and deposited strand by strand on a print bed or the previously deposited layer (see Figure 1) [4,5]. For the reinforcement with continuous fibers, adapted printer systems are used, which can extrude continuous fiber-reinforced filaments. They usually also posses a cutting unit to cut the fiber strands. Fibers made of carbon, glass, or aramid are usually used as the reinforcement material [6,7]. In addition, there are efforts to use alternative fiber materials, such as jute or flax, for additive manufacturing of composites [8,9,10].

Most of the studies in the field of continuous fiber-reinforced material extrusion focus on the determination of the mechanical properties and their influencing factors [7,10,11,12,13,14,15,16], as well as the improvement of the mechanical properties by a load-adapted fiber orientation [17,18,19,20]. It has been found that the mechanical properties of printed composites are influenced by a variety of factors. In addition to the fiber volume content, the choice of fiber material, the porosity of the components, and the fiber alignment also have a major impact on the mechanical properties. The dominant obstacles to the further spread of continuous fiber-reinforced MEX are the insufficient print quality causing high porosity, incomplete impregnation of the fibers, and the limited fiber selection with an associated limited adjustability of the mechanical properties [21]. Caminero et al. [15] attributed the low print quality and high porosity compared to conventionally produced composites to the lack of ambient pressure during production. 

### 1.2. Hybrid Composites

To address the challenge of the limited fiber selection, recent efforts have been made in order to adapt component properties for the loading case at hand by adapting a hybrid composite approach for additive manufacturing. Two or more fiber materials are combined in hybrid composites. As a result, material properties can be demonstrated that could not be achieved using a single fiber material [22,23,24]. Hybridization can be achieved by alternating layers of different fiber materials (interply), alternating different rovings in one layer (intraply), or by using pre-mixed fiber bundles (intrayarn) [22]. Previous investigations in the field of printed hybrid composites have mainly been limited to the interply approach. Haung und Joosten [25] investigated printed hybrid composites of glass and carbon fibers to obtain a pseudo-ductile material behavior. In [26], a study was presented in which the mechanical properties of printed hybrid composites consisting of aramid and carbon fiber were determined using tensile, flexural, and impact tests. The hybridization of carbon fiber-reinforced specimens with aramid fibers led to a decrease in tensile, flexural strengths, and stiffnesses, but a significant increase in impact strength. Estimation using the Rule of Hybrid Mixture (RoHM) resulted in a good agreement with the experimental data. Wang et al. [27] carried out quasi-static penetration tests with additively manufactured hybrid and non-hybrid composites of carbon and aramid fibers, respectively. A volume average stiffness model (VAS) was used to predict the stiffness and, in addition, the Rule of Hybrid Mixture was used to evaluate the hybrid effect. Hybrid tensile, flexural, and drop hammer impact tests were carried out by Zia et al. [28] using hybrid specimens manufactured with carbon and aramid fibers in an intrayarn approach. The hybrid specimens demonstrate higher strengths, stiffnesses, and energy absorption capabilities compared to non-hybrid specimens.

Recently, numerical approaches using finite element analysis (FEA) have been used to predict the mechanical performance of printed composites. Al Abadi et al. [29] used FEA to predict the mechanical properties and failure of specimens with unidirectionally oriented carbon, glass, and aramid fibers using the Hashin failure criterion. Van de Werken et al. [30] used the Tsai–Wu criterion to predict the failure of tensile specimens with curved fiber orientations under different thermal conditions. By using the FEA, the cause of failures and maximum stress could be predicted with high accuracy. Fernandes et al. [31] used both the Tsai–Wu criterion and the Hashin failure criterion to determine the failure of unidirectional carbon fiber-reinforced specimens. The two failure criteria produced very similar results with good agreement with experimental data. Extensive micro-, meso-, and macro-level modeling was performed by Polyzos et al. [32] for printed composites utilizing carbon, glass, and aramid fibers compared with experimental values from the literature. The numerical results at the macro level demonstrates a high correlation with the experimental literature values.

### 1.3. Research Gap and Aims

Since hybrid composites are still under basic investigation combined with AM, no numerical models have yet been carried out using finite element analyses. Furthermore, due to the comparative ease of implementation with the available printer systems or slicing software, only the interply and occasional intrayarn approaches have been used to produce hybrid components. Therefore, in this paper, to the best of the authors’ knowledge, the intraply approach is used for the first time to additively fabricate hybrid specimens of continuous carbon and glass fibers in a nylon matrix. The mechanical properties of these specimens are determined using tensile tests. A numerical model using a finite element analysis is developed to estimate the mechanical properties of the hybrid specimens and to draw conclusions about the failure mechanisms. The different fiber materials as well as the curved material orientations in the layers are taken into account in the developed model.

## 2. Materials and Methods

### 2.1. Printing System and Materials

**Figure 2:**
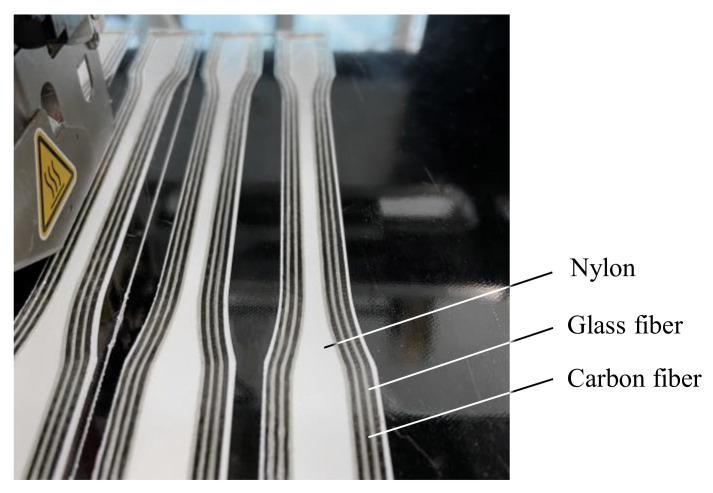
Printing process of hybrid tensile specimens with carbon and glass fibers in intraply approach.

A modified Renkforce RF2000 (Conrad Electronic SE, Hirschau, Germany) equipped with a Markforged printhead was used to fabricate the specimens (see Figure 2). The printhead allows the deposition of unreinforced thermoplastic as well as the deposition and cutting of continuous fiber-reinforced filaments. The filaments for the fabrication of the specimens were also purchased from Markforged. Nylon (“Nylon White”: polyamide 6) was used as the thermoplastic filament. Continuous fiber-reinforced filaments with carbon (C-CFF) and glass fiber reinforcement (G-CFF) were utilized as the fiber material. To prevent moisture absorption of the nylon, both the unreinforced filament and the fiber filament were stored in a Polymaker Polybox (Polymaker B.V., Shanghai, China) at a controlled humidity of < 25%. The process parameters used for the fabrication of the specimens are summarized in Table 1.

The fiber filaments contain approximately 1000 fibers embedded in a thermoplastic matrix consisting of nylon. The fiber volume content of the continuous fiber-reinforced filaments has been measured in various studies. The overview of the filaments obtained fiber volume contents is given in Table 2. 

### 2.2. Specimen Design and Fabrication 

**Figure 3:**
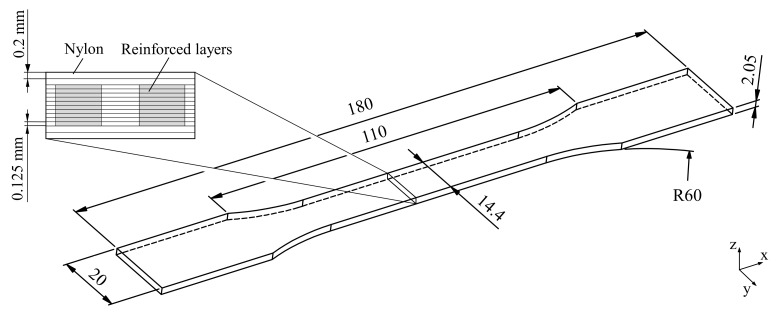
Dimensions of the tensile specimen with the schematic layer structure.

In this paper, both hybrid and non-hybrid tensile specimens were manufactured from nylon with carbon and glass fiber reinforcement. The specimen design is based on DIN EN ISO 527-4 type 1B [36]. The dimensions can be seen in Figure 3. They were printed with 10 fiber-reinforced layers with a layer thickness of 0.125 mm. In addition, the specimens were printed with two unreinforced bottom and top layers of nylon with a layer thickness of 0.2 mm.

**Figure 4:**
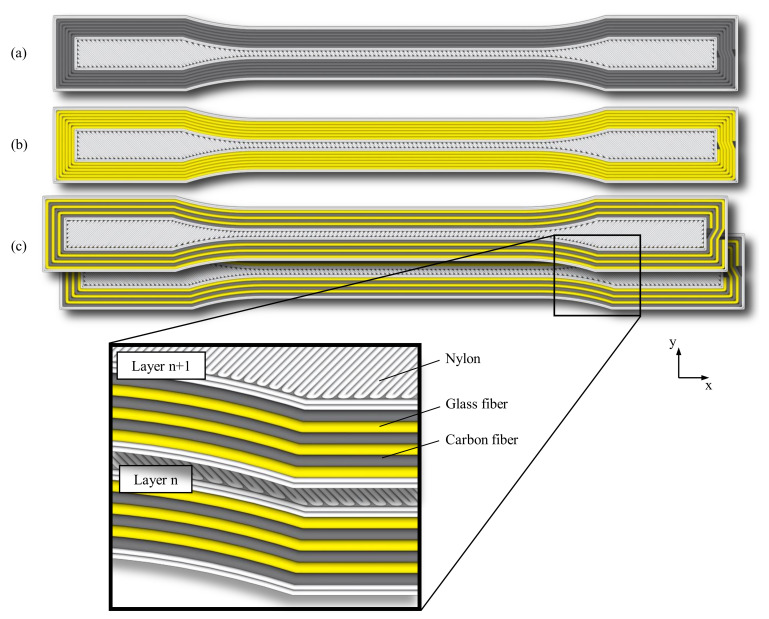
Schematic fiber orientation of the non-hybrid tensile specimens: (**a**) with carbon fiber reinforcement, (**b**) with glass fiber reinforcement, (**c**) schematic fiber orientation, and the layered structure of the hybrid tensile specimens with carbon and glass fibers.

**Figure 5:**
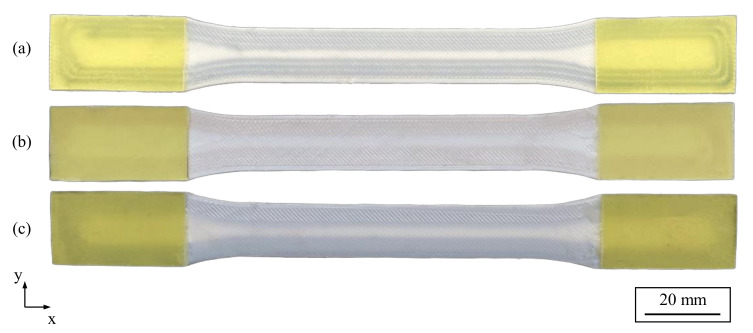
Specimens with tabs made of glass fiber-reinforced plastic: (**a**) hybrid tensile specimen, (**b**) tensile specimen with glass fiber reinforcement, and (**c**) tensile specimen with carbon fiber reinforcement.

Five specimens were manufactured and tested for each specimen configuration. The G-code, a machine code readable by 3D printers, was created using a self-written slicing program in Rhino 7, supported by the visual programming language Grasshopper. The fibers were positioned following the outer contours of the tensile specimens to obtain a multiaxial stress state. In the hybrid specimens, the fiber materials alternate from contour path to contour path, resulting in a 50% proportion of carbon and glass fibers each (see Figure 4c). The cutting positions were determined in a controlled manner at the end of the tensile specimen in the unstressed areas outside the gauge length. The unreinforced areas were filled with nylon. During the production of the hybrid tensile specimens, the fiber material was changed manually after the completion of the respective fiber paths. Following the underlying standard [36], the end sections of the tensile specimens were provided with tabs made of glass fiber-reinforced plastic with an orientation of ±45°. The tabs were bonded using 3M Scotch-Weld DP 810 (3M, Saint Paul, MN, USA), a highly elastic adhesive (see Figure 5). The fiber volume fraction of the tensile specimens is calculated according to the following formula:

(1) $$V_{f}=\frac{v_{f}}{V_{t}}\times 100\%$$

where *V*_f_ represents the fiber volume fraction of the entire specimen, *v*_f_ is the volume of the fibers in the entire specimen, and *V*_t_ is the total volume of the specimen. Here, the fiber volume fraction represents the ratio of the volume of the fibers to the total volume of the component. It should be noted that components manufactured by MEX usually exhibit high porosity. However, the porosity of the component is not taken into account in the equation used. For the calculation, a fiber volume fraction of the filaments of 34% was assumed. The relative and absolute fiber volume fractions are listed in Table 3.

### 2.3. Mechanical and Microstructural Characterization

Tensile tests were carried out to determine the mechanical properties. The tensile tests were carried out according to DIN EN ISO 527-4 [36] and the specimens were prepared according to the underlying standard. The Zwick/Roell Z100 universal testing machine (ZwickRoell GmbH & Co. KG, Ulm, Germany) was used for testing. The specimens were clamped into the testing machine with the entire grip section. The test speed was 2 mm/min for all tests. Specimen elongation was determined via an extensometer with a gauge length of 100 mm and force was measured via a 100 kN load cell. All tensile tests were performed under identical environmental conditions with a constant temperature of ~23 °C. The stresses, strains, and stiffnesses were calculated based on the underlying standard [36]. For the investigation of the fracture surfaces, images were taken using a scanning electron microscope (SEM). The Zeiss Leo 1455VP (Carl Zeiss AG, Oberkochen, Germany) with an accelerating voltage of 10 kV was used.

### 2.4. FE-Modeling

To better understand the damage initiation and to predict the failure load and the stiffness of the specimens, a numerical model was created using the FE software ABAQUS (Version 2020). The main challenge in modeling additively manufactured composite components is assigning direction-dependent material properties to partially complex fiber paths. To generate an FE model with complex fiber paths, a layer of the specimen was first loaded into the FE software (Abaqus version 2020 )as a shell element. The generation of complex fiber paths in the self-written slicing software allowed the direct transfer of the fiber path geometries into the FE software. Using the layer geometry and the fiber trajectories, different sections were generated to which separate material properties of the fibers or the matrix can be assigned. For the numerical calculation, the mechanical parameters listed in Table 4 were used, which are described in the literature and manufacturer data sheets, determined in preliminary tests according to the respective DIN standards, or were estimated.

**Figure 6:**
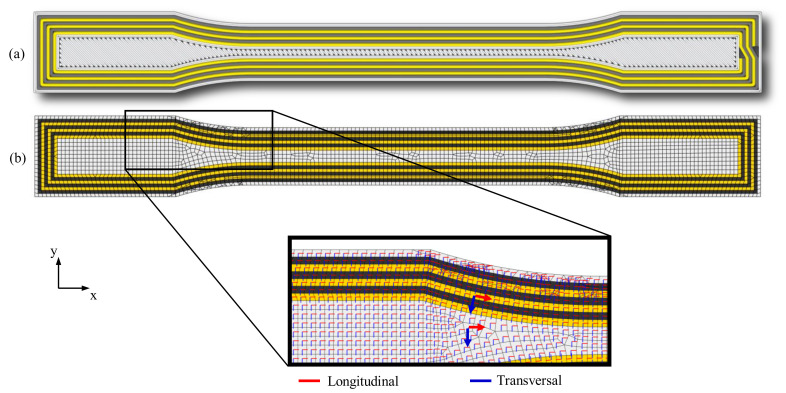
Comparison between the following: (**a**) generated tool paths in the slicing software and (**b**) a modeled fiber-reinforced layer with the longitudinal and transverse orientation of the materials with red and blue arrows, respectively.

**Figure 7:**
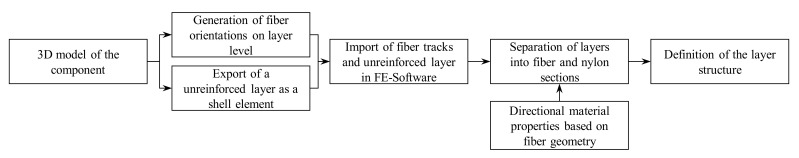
Approach to the creation of the FE model of a printed component with complex fiber trajectories.

For simplification, the unreinforced areas were assumed to be an isotropic material. In addition, direction-dependent material properties were assigned to the fiber areas based on the fiber path geometry. This procedure was repeated for each different layer (see Figure 6). In the final step, the layers were linked together via a tie constraint to create a complete geometry (see Figure 7).

**Figure 8:**
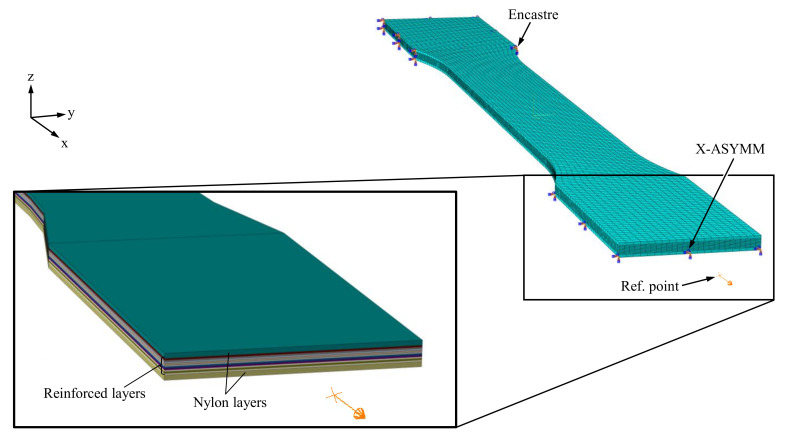
Meshing and constraint conditions of the FE model with a section of the FE shell model with the assigned thicknesses of the shell elements.

For the application of the Hashin and Tsai–Wu failure criterion, planar stress elements and shell elements are used in Abaqus. In addition, the component geometry is in the form of plane shells in the self-written slicing software. Therefore, the use of shell elements in Abaqus allowed a direct transfer of the part geometry from the slicing software to the FE software. The shells were assigned thicknesses corresponding to the layer thickness of the printed specimens (see Figure 8). Quadrilateral general-purpose composite (S4R) shell elements with reduced integration were used to mesh the model. The efficient S4R elements feature four nodes with one integration point. The results were provided in the respective integration points of the mesh. The use of three-dimensional elements was omitted since only plane stress states are considered. After a convergence study for the fiber-reinforced and non-reinforced layers, an average element size of 1 mm was specified with a minimum size control of 0.1. This resulted in 4120 elements with 4257 nodes each in the fiber-reinforced layers and 3620 elements with 3820 nodes each in the non-reinforced bottom and top layers. For the simulation of the tensile test, one side of the tensile specimen was clamped, while the opposite side was assigned only one degree of freedom in the x-direction. The displacement was initiated by a reference point associated with the displaceable side of the specimen. The total force was also measured at the reference point (see Figure 8).

### 2.5. Failure Criteria

Damage initiation was determined using the Hashin [38,39] and Tsai–Wu failure criteria [40], which were implemented in ABAQUS. Unlike the polynomial Tsai–Wu criterion, the Hashin failure criterion distinguishes between four different failure modes. These are subdivided into damage initiation by fiber tension ($F_{f}^{t}$), fiber compression ($F_{f}^{c}$), matrix tension ($F_{m}^{t}$), and matrix compression ($F_{m}^{c}$). The Hashin failure criterion is particularly interesting because studies [20] in the context of printed composites have proved that fibers oriented transversely to the load direction can promote fracture at the respective fiber strands. The four failure modes are expressed by Equations (2)–(5) [38,39]. 

Failure due to fiber tension (*σ*_11_ ≥ 0):

(2) $$F_{f}^{t}={\left(\frac{\sigma_{11}}{X_{T}}\right)}^{2}=\;\left\{\;\begin{array}{c} \geq 1,\;Failure\;\;\;\\ \;\;<1,\;No\;failure \end{array}\right.$$

Failure due to fiber pressure (*σ*_11_ < 0):

(3) $$F_{f}^{c}={\left(\frac{\sigma_{11}}{X_{C}}\right)}^{2}=\;\left\{\;\;\begin{array}{c} \geq 1,\;Failure\;\;\;\\ \;\;<1,\;No\;failure \end{array}\right.$$

Failure due to matrix tension (*σ*_22_ ≥ 0):

(4) $$F_{m}^{t}={\left(\frac{\sigma_{22}}{Y_{T}}\right)}^{2}+{\left(\frac{\tau_{12}}{S_{L}}\right)}^{2}=\;\left\{\;\begin{array}{c} \geq 1,\;Failure\;\;\;\\ \;\;<1,\;No\;failure \end{array}\right.$$

Failure due to matrix pressure (*σ*_22_ < 0):

(5) $$F_{m}^{c}={\left(\frac{\sigma_{22}}{2S_{T}}\right)}^{2}+\left[{\left(\frac{Y_{C}}{2S_{T}}\right)}^{2}-1\right]\frac{\sigma_{22}}{Y_{C}}+{\left(\frac{\tau_{12}}{S_{L}}\right)}^{2}=\;\left\{\;\begin{array}{c} \geq 1,\;Failure\;\;\;\\ \;\;<1,\;No\;failure \end{array}\right.$$

where *σ*_11_, *σ*_22_, and *τ*_12_ are the components of the plane stress tensor. *X*_T_ and *X*_C_ are the tensile and compressive strength of the composite in the longitudinal direction. *Y*_T_ and *Y*_C_ are the tensile and compressive strength in the transverse direction. *S*_T_ and *S*_L_ describe the shear strength in the longitudinal and transverse directions. The influence of shear stress on fiber tensile failure is considered negligible.

The Tsai–Wu criterion is a simpler approach and, unlike the Hashin failure criterion, it does not distinguish between different failure modes, but only calculates the first occurrence of a failure in the composite. For plane stress problems, failure occurs according to the Tsai–Wu criterion when the following equation is violated:

(6) $$F_{1}\sigma_{11}+F_{2}\sigma_{22}+F_{11}\sigma_{11}^{2}+F_{22}\sigma_{22}^{2}+2F_{12}\sigma_{11}\sigma_{22}+F_{66}\tau_{12}^{2}<1$$

*F*_1_, *F*_2_, *F*_11_, *F*_22_, *F*_12_, and *F*_66_ are coefficients that can be calculated according to the equations given in Table 5. 

Here, $F_{12}^{*}$ is an interaction coefficient and has a validated range from –1 ≤ $F_{12}^{*}$ ≤ 1. Ideally, the coefficient is determined by biaxial material tests. However, biaxial tests are difficult to execute and there are no standard methods for determining the coefficient. Narayanaswami and Adelman [41] suggest the coefficient be set to zero, arguing that there are no significant interactions between longitudinal and transverse stresses. This is supported by studies demonstrating that the value of *F*_12_ is relatively insignificant for the accuracy of the results and so, it is often set to zero [41,42]. As a consequence, this paper considers the discussed value of zero.

## 3. Results and Discussion

### 3.1. Results from Experimental Tensile Tests

**Figure 9:**
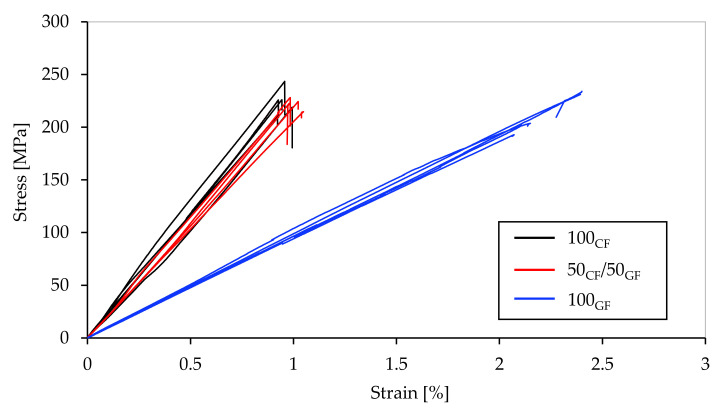
Stress–strain curves of the tensile tests.

The tensile tests were performed in accordance with the procedure described in Section 2.3. The stress–strain curves recorded during the tests are presented in Figure 9. Table 6 summarizes the characteristic values of the tensile tests obtained experimentally. Brittle failure was observed in all of the specimen configurations. 

Upon comparing the experimental data, it can be observed that the non-hybrid carbon fiber-reinforced specimens exhibited the highest stiffness (23.8 GPa ± 1.2 GPa) and tensile strength (225.1 MPa ± 11.4 MPa) among the three specimen configurations. In contrast, the specimens reinforced with glass fibers displayed significantly lower stiffness, with a Young’s modulus of 10.2 GPa ± 0.6 GPa. However, the tensile strength of the glass fiber-reinforced specimens was only slightly lower, at 212.9 MPa ± 16.9 MPa. Remarkably, the hybrid specimens with 50% carbon fiber reinforcement and 50% glass fiber reinforcement (relative fiber volume fraction) exhibited very high stiffness values. The hybrid specimens displayed a stiffness of 21.9 GPa ± 1.2 GPa, which was comparable to the carbon fiber-reinforced specimens, despite the 50% share of less stiff glass fibers. The tensile strength of the hybrid test specimens was between the values of the carbon fiber and glass fiber-reinforced specimens, at 220.2 MPa ± 6.3 MPa. This is particularly remarkable considering the very different tensile strengths of the carbon and glass fibers. The small influence of the longitudinal tensile strengths of the fiber material on the failure of the specimens can be explained by the multiaxial loads in the shoulder region. As a result, material parameters such as shear strength and transverse tensile strength also have a major influence on the strength. This can be attributed to the fact that significantly stiffer carbon fibers fail when the specific yield strength is exceeded, resulting in a total failure of the entire specimen, even if the yield strength of the less stiff glass fibers has not yet been reached. In contrast, the experimentally determined average failure strain of the glass fiber-reinforced specimens was 2.1% ± 0.14%, which was significantly higher than the values of the other two specimen types.

**Figure 10:**
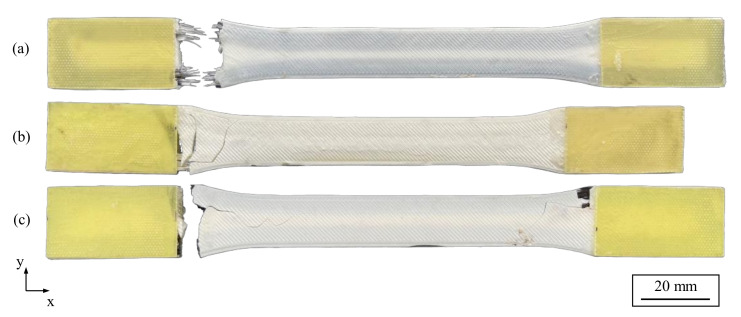
Examples of fractured tensile specimens: (**a**) with hybrid fiber reinforcement, (**b**) with glass fiber reinforcement, and (**c**) with carbon fiber reinforcement.

Figure 10 shows an example of a fractured specimen for each specimen type and the fracture patterns were consistent across all specimen types. It is apparent that all specimens failed in the shoulder area, which is attributed to the curved fiber paths and the resulting transverse loads to the longitudinal direction of the fibers. This leads to critical shear stresses that promote premature failure of the tensile specimens, even though the longitudinal tensile strength of the fibers has not been reached. Futhermore, the relatively low fiber volume content of about 12.4% in the specimens contributes to the relatively low tensile strength. Consequently, dog-bone tensile specimens with contour-based fiber orientation are unsuitable for determining the mechanical properties of continuous fiber-reinforced printed specimens under a tensile load. Instead, unidirectionally reinforced specimens should be used to determine the tensile strength or Young’s modulus. However, in this study, contour-based fiber orientations were deliberately chosen to experimentally and numerically investigate the influence of multiaxial loading, which is not present in unidirectional fiber orientations.

### 3.2. FEA Results from Tensile Model

To draw conclusions regarding the failure initiation, numerical calculations were performed for the tensile tests using both the Hashin and Tsai–Wu failure criteria as failure criteria. Table 7 compares the experimentally measured failure loads of the tensile tests, along with their respective standard deviations, with those predicted based on the Tsai–Wu and Hashin criteria. In this paper, it was assumed that failure occurs at a limit value of ≥1. The errors of the theoretical values calculated by FEA are determined according to the following equation:

(7) $$\%\;Error=\left|\;\frac{theoretical-experimental}{experimental}\;\right|\times 100\%$$

**Figure 11:**
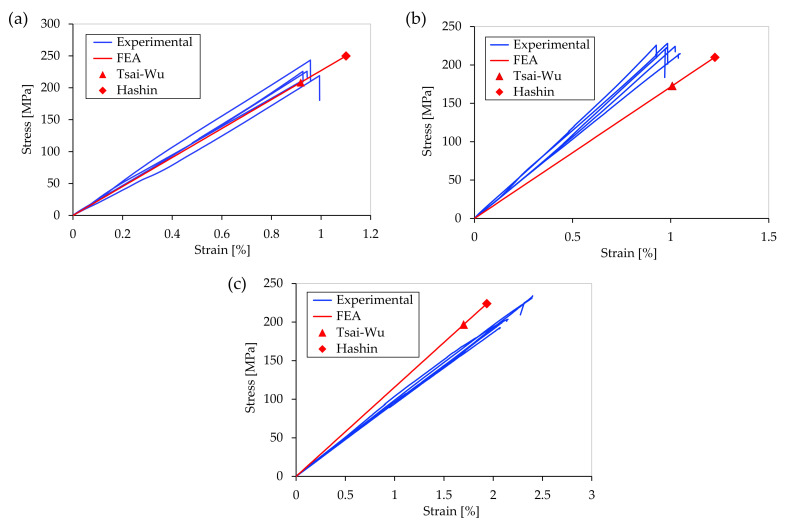
Experimental and numerically calculated stress–strain curves of the tensile tests: (**a**) with carbon fiber reinforcement, (**b**) with hybrid fiber reinforcement, and (**c**) with glass fiber reinforcement.

Figure 11 shows the stress–strain curves of the tensile tests and the numerically calculated curves together with the points of failure according to the Hashin and Tsai–Wu failure criteria. A comparison of the experimental with the theoretical values reveals a high degree of agreement, especially for the maximum failure load. Both the Hashin and Tsai–Wu failure criteria were able to predict the failure relatively accurate. An exception in this context is the significantly too low prediction values of the hybrid specimen with the Tsai–Wu failure criterion, with a deviation of 21.5%. The average deviation of the maximum failure load using the Tsai–Wu criterion is about 12.3%, while the average deviation of the Hashin failure criterion is about 6.9%. The partially high discrepancies between the predicted values according to the Hashin and Tsai–Wu criteria are remarkable. In all cases, the Tsai–Wu criterion leads to values that are significantly lower than the experimentally determined values. This may be due to high shear stresses and tensile stresses in the longitudinal and transverse directions of the fibers. These are considered separately in the Hashin failure criterion, whereas in the Tsai–Wu failure criterion, all the quantities are considered together. 

**Figure 12:**
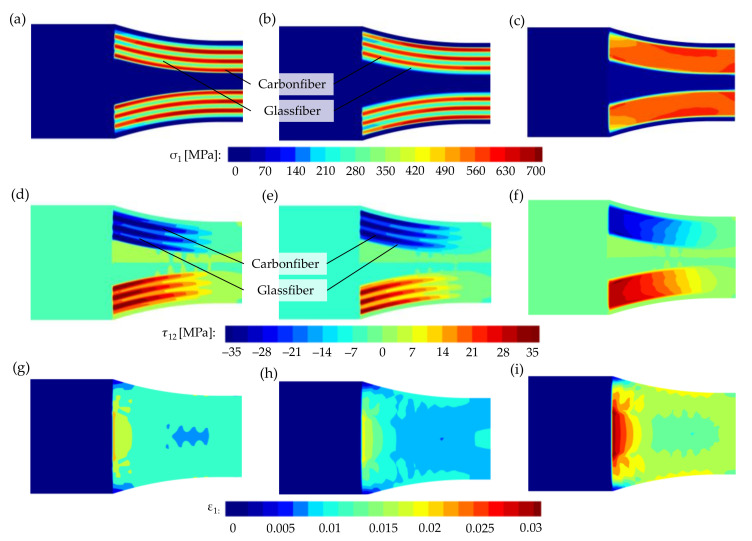
Normal stresses in the longitudinal direction σ_1_: (**a**,**b**) hybrid specimens with different fiber orientations, (**c**) carbon fiber-reinforced specimen; shear stresses τ_12_: (**d**,**e**) hybrid specimen with different fiber orientations, (**f**) carbon fiber-reinforced specimen; strains in longitudinal direction ε_1_: (**g**) hybrid specimen, (**h**) carbon fiber-reinforced specimen, and (**i**) glass fiber-reinforced specimen.

**Figure 13:**
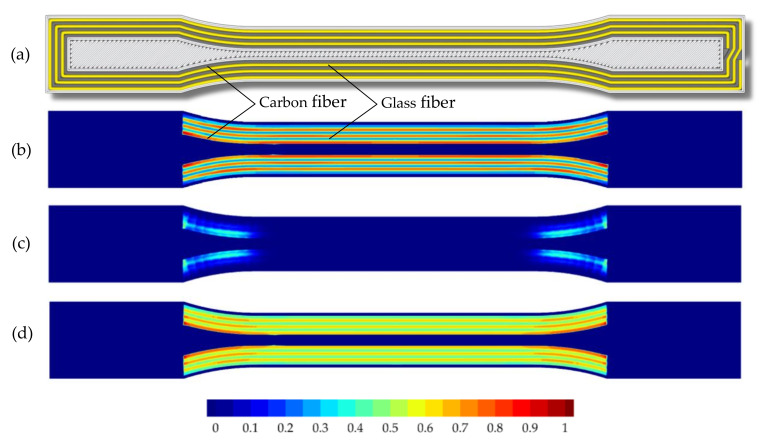
Comparison between damage predictions for hybrid tensile specimen (50_CF_/50_GF_): (**a**) visualization of G-code, (**b**) Hashin damage criterion–fiber tension $F_{f}^{t}$, (**c**) Hashin damage criterion–matrix tension $F_{m}^{t}$, and (**d**) Tsai–Wu criterion; Hashin and Tsai–Wu damage criterion, respectively, when reaching a value of ≥1.

**Figure 14:**
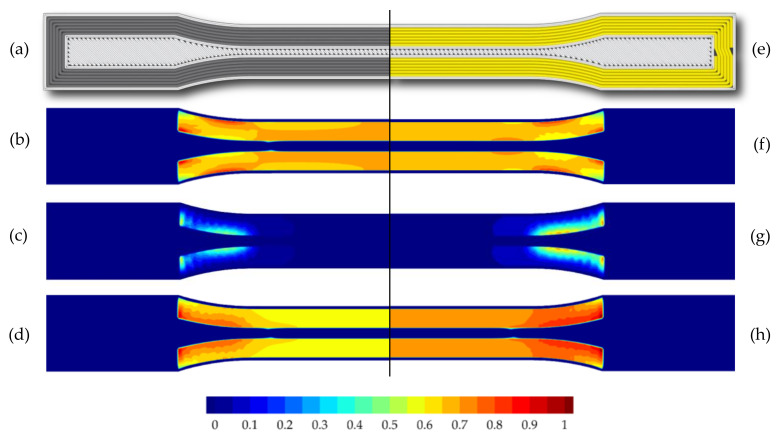
Comparison between damage predictions for the non-hybrid tensile specimen with carbon fiber reinforcement (100_CF_): (**a**) visualization of the G-code, (**b**) Hashin damage criterion–fiber tension $F_{f}^{t}$, (**c**) Hashin damage criterion–matrix tension $F_{m}^{t}$, and (**d**) Tsai–Wu criterion; for the non-hybrid tensile specimen with glass fiber reinforcement (100_GF_): (**e**) visualization of the G-code, (**f**) Hashin damage criterion–fiber tension $F_{f}^{t}$, (**g**) Hashin damage criterion–matrix tension $F_{m}^{t}$, and (**h**) Tsai–Wu criterion; Hashin and Tsai–Wu damage criterion, respectively, when reaching a value of ≥ 1.

In Figure 12 the different types of shoulder region loading are illustrated using an FEM analysis. The two fiber types in the hybrid specimens show significant differences in normal stresses (Figure 12a,b) and shear stresses (Figure 12d,e). This can be attributed to the different stiffness of carbon and glass fibers. In this case, the carbon fibers are significantly more loaded at lower strain rates. No significant differences between the various fiber layouts, which are determined by the order of the fiber strands, are evident in Figure 12a,b or in Figure 12d,e. Furthermore, it is apparent that the fibers bear the majority of the load, with the surrounding nylon matrix contributing only to the force transmission and stabilization of the fiber strands. From the strains in Figure 12g–i, it can be inferred that the strains of the glass fiber-reinforced specimens are greater compared to the hybrid and carbon fiber-reinforced specimens. This is due to the lower stiffness of the glass fibers. Greater strains of the fiber-reinforced regions are particularly observed on the inner sides of the strands, which align with damage predictions based on Hashin and Tsai–Wu criteria (see Figure 13 and Figure 14). Furthermore, it is apparent that the nylon matrix between the fiber strands in the shoulder region experiences high levels of deformation due to the load-bearing behavior of the fibers. However, since unreinforced nylon has a significantly lower stiffness than the fibers, the different deformations could promote a lateral debonding of the strands from the nylon matrix. This could also explain the cracks in the specimens shown in Figure 10.

The numerical calculations demonstrate that only the two failure modes, fiber tension and matrix tension, assume larger values (see Table 8). Therefore, in addition to the Tsai–Wu failure indices, only the Hashin failure after fiber tension and after matrix tension is shown in Figure 13 and Figure 14.

The different material properties of the fibers in the hybrid specimens also result in different predictions for the time of failure. According to both Hashin and Tsai–Wu, the carbon fibers fail first due to their higher stiffness. The failure of the non-hybrid carbon and glass fiber-reinforced specimens (see Figure 14) results in very similar failure indices. It can be observed that both the Hashin failure criterion and the Tsai–Wu failure criterion reliably predict the critical component locations. According to both Hashin fiber tension and matrix tension criteria, the fiber strands in the inner shoulder region fail first. This combination of loads and failure modes also leads to a constant fracture pattern at the predicted locations during the experimental tensile tests. The fracture initiation at the inner fiber strands is in addition supported by the fracture patterns of the specimens. Cracks in the glass- and carbon fiber-reinforced specimens run from the inner fiber areas toward the center of the specimen (see Figure 10).

From a design point of view, the precise determination of the occurring failure modes according to Hashin represents an interesting opportunity for optimization in the context of continuous fiber-reinforced MEX. A great potential of continuous fiber-reinforced MEX is the variable fiber deposition. High transverse tensile loads or even shear stresses can be effectively countered by an alternative layer layout with an adapted fiber orientation and adapted fiber selection. 

### 3.3. Hybrid Effect

The mechanical properties of hybrid composites can be estimated based on the RoHM equations [43]. The mechanical properties of hybrid composites are calculated based on the two fiber composite systems according to the following equation:

(8) $$E_{hyb}=E_{c1}\times V_{c1}+E_{c2}\times (1-V_{c1})$$

where *E*_*c*1_ and *E*_*c*2_ are Young’s moduli of the first and second fiber composite systems, respectively. *V*_*c*1_ and *V*_*c*2_ are the relative volume fractions of the first and second fiber composite systems, respectively, in the total composite. The total volume is calculated from both volume fractions according to the following equation:

(9) $$V_{t}=V_{c1}+V_{c2}=1$$

where *V_t_* is the volume of the entire composite. Larger positive or negative deviations from the values calculated utilizing RoHM equations are called positive or negative hybrid effects in this context and can be determined according to the following equation (exemplified by the Young’s modulus) [44]:

(10) $$\lambda_{E}=\frac{E_{exp}}{E_{hyb}}-1$$

*λ_E_* thus stands for the deviation of the experimentally determined Young’s modulus *E_exp_* from the value *E_hyb,_* predicted utilizing the RoHM equations. A positive value of *λ_E_* thus represents a positive hybrid effect, while a negative value of *λ_E_* stands for a negative hybrid effect.

**Figure 15:**
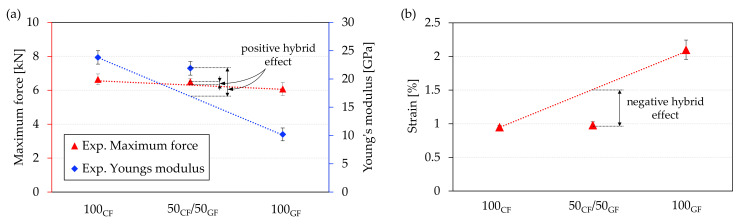
Positive and negative hybrid effects: (**a**) maximum force and Young’s modulus and (**b**) failure strain.

The experimentally determined average Young’s modulus of the hybrid specimens was significantly higher than the values calculated through the RoHM equations. For Young’s modulus of the hybrid specimens determined using RoHM, there was a theoretical value of approximately 17 GPa. However, the actual measured value is 21.9 GPa ± 1.2 GPa, resulting in a hybrid effect of 28.8%. In contrast, no significant hybrid effect can be observed for the failure load. The experimental value of 6.5 kN ± 0.19 kN is very close to the theoretically calculated value of 6.47 kN. For the failure strains of the hybrid specimens, on the other hand, there is a negative hybrid effect. At only 0.98%, the failure strain of the hybrid specimens is only marginally higher than that of the carbon fiber-reinforced specimens at 0.95%. The reason for this is that if the material’s failure strain is surpassed and individual carbon fiber strands fail, the component is significantly weakened, leading to an immediate, catastrophic failure. The hybrid effects for the hybrid specimens tested are illustrated in Figure 15. Rajpurohit et al. [44] also determined a negative hybrid effect for the failure strain for conventional intraply specimens with glass and carbon fiber reinforcement, whereas specimens in the interply approach resulted in an improvement in the failure strain. Additively manufactured tensile specimens in the interply approach [26] and intrayarn approach [28] also showed improvements in failure strains. This could be related to a limitation of damage propagation in the carbon fiber layers in the case of specimens fabricated using the interply approach, as suggested by Rajpurohit et al. [44]. The improved failure strain of specimens fabricated using the intrayarn approach could be caused by the improved dispersion of the fibers.

### 3.4. Microstructural Analysis

**Figure 16:**
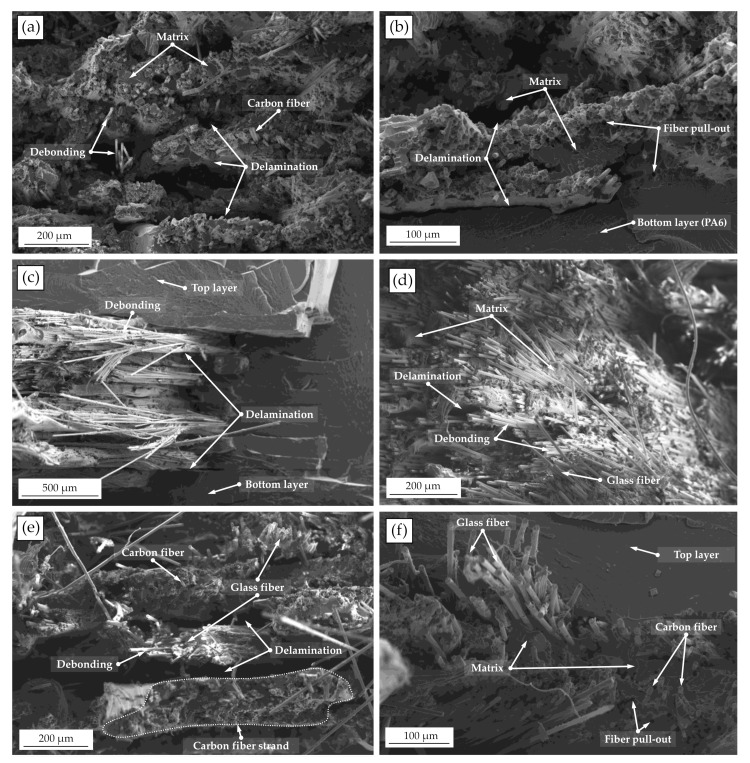
SEM images of the fracture surfaces: (**a**,**b**) carbon fiber-reinforced specimens, (**c**,**d**) glass fiber-reinforced specimens, and (**e**,**f**) hybrid specimens.

To understand the specimens failure causes and possible differences in failure modes between the two composite systems, images of the fracture surfaces were taken using a SEM. The fracture surfaces of the hybrid and non-hybrid specimens are shown in Figure 16. 

In the cross-sectional images, irregularly distributed fibers with dense polymer areas are noticeable, which can lead to non-uniform stresses in the fiber-reinforced areas. Non-uniformly distributed fibers have been described in several studies [20,30,34]. This can be attributed to the fiber filament’s low fiber volume content of 31.5–38.8%. Based on the fracture patterns, it is also evident that significantly stronger debonding occurs in the glass fiber-reinforced specimen areas compared with the carbon fiber-reinforced areas. This manifests itself in exposed fibers without a surrounding matrix, which indicates poor impregnation. In addition, the shear stresses that occur and the loading under transverse tension could increase the debonding. The simulations and the high value for matrix tensile according to Hashin also suggest high loads transverse to the fiber. The carbon fiber-reinforced areas show brittle, broken fibers, most of which are still bonded to the surrounding matrix. Sporadic fiber pull-outs and exposed fibers can also be seen here. Delamination between the layers can be observed in both types of reinforcement. This is a known cause of failure in conventional composites, which is exacerbated by the layer-by-layer manufacturing process in printed composites. Several studies have also identified debonding and delamination of printed specimens as major failure mechanisms [15,45,46]. In particular, strong delamination is observed between layers in hybrid specimens (see Figure 16e,f). This is due to the alternating fiber strands printed side by side or on top of each other with strongly different stiffnesses (see Figure 16e). If the failure strain of the carbon fibers is exceeded, the detachment could lead to an abrupt breakage of the carbon fiber strands and to a simultaneous slippage of the glass fiber strands, which are still intact for a short time. The low bond adhesion between the strands can also explain the only slightly higher experimentally measured strain rates of the hybrid specimens compared with the carbon fiber specimens. A better dispersion might be necessary for a significant improvement in the strain rates.

## 4. Conclusions

In this paper, a novel approach was presented to fabricate hybrid composites in an intraply approach using continuous fiber-reinforced MEX. Tensile specimens were fabricated with carbon and glass fiber reinforcement in a nylon matrix. The mechanical properties of non-hybrid carbon and glass fiber-reinforced and hybrid tensile specimens, with 50% glass and carbon fiber content, respectively, were manufactured and tested. In addition, an FE model of the hybrid and non-hybrid specimens was created, taking into account the unreinforced component regions, curved fiber orientations, and the direction-dependent fiber properties. The failure of the specimens was determined using the Hashin and Tsai–Wu failure criteria. The experimental data were compared with the values calculated numerically based on the FEA and those calculated based on the Rule of Hybrid Mixture. The main findings can be summarized as follows:

- Fiber orientation is crucial for maximizing mechanical properties; particularly shear stresses and transverse tensile stresses should be minimized.
- The primary failure mechanisms included delamination and debonding. Debonding was significantly more apparent in the glass fiber areas due to poor impregnation and consequent low bond strength of the fibers with the matrix. In the hybrid specimens, strong delaminations between the different fiber strands dominated, which can be attributed to the strongly different stiffnesses.
- The carbon fiber-reinforced specimens showed the highest stiffnesses and strengths, followed by the hybrid specimens. The glass-fiber-reinforced specimens exhibited the lowest tensile strengths and stiffnesses. The hybrid tensile specimens showed positive hybrid effects in terms of stiffness with 28.8%. The strength, on the other hand, did not show any hybrid effect, while the failure strains were only slightly higher than those of the carbon fiber-reinforced specimens.
- Using both the Hashin and Tsai–Wu failure criteria, the maximum failure loads of the hybrid and non-hybrid specimens could be predicted with good accuracy. The positions of specimen failure were also predicted reliable.
- The FE modeling described in this paper can be applied to the modeling of printed hybrid components as well as to the modeling of components with a complex fiber orientation. Nevertheless, this involves a comparatively high manual effort.
- To avoid increasing manufacturing time for printed hybrid composites with an interply or intraply approach, which is accompanied by a regular change of the fiber material, the existing printer systems have to be extended by another fiber print head.

Further research in the area of additively manufactured hybrid composites should include comparative studies that consider the different hybridization modes, such as interply, intraply, and intrayarn. In addition, different combinations of materials should be included. Furthermore, interfaces between slicing software for continuous fiber-reinforced components and FE applications should be developed to reduce the manual effort involved in reproducing the often complex fiber paths. Furthermore, design methods have to be developed which, in addition to homogeneous hybridization in the layer plane, also exploit the design freedom of continuous fiber-reinforced MEX by printing entire component areas with variably adaptable fiber materials. This could enable completely new component properties with variable stiffness, strength, and impact resistance.

## Figures and Tables

**Table 1:** Used process parameters to manufacture the specimens.

	Parameter	Value	Unit
General	Fiber/filament extruder temperature	270	°C
	Print bed temperature	95	°C
	Infill density	100	%
Nylon	Nylon filament diameter	1.75	mm
	Strand width	0.4	mm
	Nylon printing speed	40	mm/s
	Nylon infill angle	+45/−45	°
	Bottom and top layers’ height	0.2	mm
	Number of bottom/top layers	2	−
Fiber	C-CFF/G-CFF diameter	0.4	mm
	Fiber strand width	0.9	mm
	Fiber printing speed	20	mm/s
	Layer height fiber/nylon	0.125	mm

**Table 2:** Fiber volume contents of fiber filaments given in the literature.

Reference	Carbon (C-CFF)	Glass (G-CFF)
Van der Klift [11]	34.5%	−
Dutra [33]	32.8%	−
Chabaud [34]	35%	38.8%
Pascual-Gonzáles [35]	33.9–36.4%	31.5–38%

**Table 3:** Relative and absolute fiber volume contents of manufactured hybrid and non-hybrid specimens.

Specimen	Relative Fiber Volume Content		Absolute Fiber Volume Content	
	Carbon	Glass	Carbon	Glass
100_CF_	100%	0%	12.4%	–
50_CF_/50_GF_	50%	50%	6.2%	6.2%
100_GF_	0%	100%	–	12.4%

**Table 4:** Assumed mechanical properties of the materials used.

Property	C-CFF [30,37]	G-CFF [37]	Nylon [30,37]
Density, *ρ* (g/cm^3^)	1.2	1.5	1.1
Longitudinal elastic modulus, *E*_1_ (MPa)	52,000	25,000	940
Transverse elastic modulus, *E*_2_ (MPa)	4000	2500	940
Shear modulus, *G*_12_ and *G*_23_ (MPa)	2000	1400	340
Poisson’s ratio, *v*_12_	0.33	0.37	0.4
Longitudinal tensile strength, *X_T_* (MPa)	700	548	53.8
Longitudinal compressive strength, *X_C_* (MPa)	320	118	53.8
Transverse tensile strength, *Y_T_* (MPa)	48	34	53.8
Transverse compressive strength, *Y_C_* (MPa)	100	56	53.8
Longitudinal shear strength, *S_L_* (MPa)	73	67	68.9
Transverse shear strength, *S_T_* (MPa)	73	67	68.9

**Table 5:** Equations for calculating the coefficients for the Tsai–Wu criterion.

*F_1_*	*F_2_*	*F_11_*	*F_22_*	*F_12_*	*F_66_*
$\frac{1}{X_{T}}-\frac{1}{X_{C}}$	$\frac{1}{Y_{T}}-\frac{1}{Y_{C}}$	$\frac{1}{X_{T}X_{c}}$	$\frac{1}{Y_{T}Y_{c}}$	$\frac{F_{12}^{*}}{\sqrt{X_{T}X_{c}Y_{T}Y_{c}}}$	$\frac{1}{S^{2}}$

**Table 6:** Experimentally determined mechanical properties of the hybrid and non-hybrid specimens with the average, minimum and maximum values and standard deviations (SD); the tensile strength is related to the cross-section in the range of the gauge length.

Specimen	Maximum Force (kN)				Tensile Strength (MPa)				Elastic Modulus (Gpa)			
	Avg.	Min.	Max.	SD	Avg.	Min.	Max.	SD	Avg.	Min.	Max.	SD
100_CF_	6.64	6.23	7.19	0.32	225.1	208.6	243.5	11.4	23.8	22.1	25.5	1.2
50_CF_/50_GF_	6.5	6.23	6.74	0.19	220.2	211.1	228.2	6.3	21.9	20.5	23.5	1.2
100_GF_	6.29	5.7	6.94	0.5	212.9	192.9	235.3	16.9	10.2	9.3	12.4	0.6

**Table 7:** Comparison between the experimental and the tensile test results predicted by Tsai–Wu and Hashin failure criterion with the relative errors.

Specimen	Maximum Failure Load (kN)			Young’s Modulus (GPa)	
	Experimental	Tsai–Wu (FEA)	Hashin (FEA)	Experimental	FEA
100_CF_	6.64 ± 0.32	6.14 (7.5%)	7.37 (11%)	23.8 ± 1.2	22.7 (4.6%)
50_CF_/50_GF_	6.5 ± 0.19	5.1 (21.5%)	6.2 (4.6%)	21.9 ± 1.2	17.4 (20.5%)
100_GF_	6.29 ± 0.5	5.8 (7.8%)	6.61 (5.1%)	10.2 ± 1.1	11.6 (13.7%)

**Table 8:** Damage predictions for the hybrid and non-hybrid specimens according to the Hashin failure criterion at maximum failure load.

Specimen	Fiber Tension ($F_{f}^{t}$)	Matrix Tension ($F_{m}^{t}$)	Fiber Compression ($F_{f}^{c}$)	Matrix Compression ($F_{m}^{c}$)
100_CF_	1	0.81	0	0.05
50_CF_/50_GF_	1	0.86	0	0.1
100_GF_	1	0.97	0	0.05

## Data Availability

The data presented in this study are available in the article.

## Glossary

Abbreviation	Meaning
AM	Additive manufacturing
C-CFF	Carbon continuous fiber-reinforced filament
CF	Carbon fiber
*E*_1_	Longitudinal elastic modulus
*E*_2_	Transverse elastic modulus
*E*_*c*1_	Young’s modulus of the first fiber composite system
*E*_*c*2_	Young’s modulus of the second fiber composite system
*E_exp_*	Experimentally determined Young’s modulus
*E_hyb_*	Young’s modulus of the hybrid composite
FDM	Fused Deposition Modeling
FEA	Finite element analysis
FFF	Fused Filament Fabrication
$F_{f}^{c}$	Fiber compression (Hashin)
$F_{f}^{t}$	Fiber tension (Hashin)
$F_{m}^{c}$	Matrix compression (Hashin)
$F_{m}^{t}$	Matrix tension (Hashin)
*G*_12_, *G*_23_	Shear modulus
G-CFF	Glass continuous fiber-reinforced filament
GF	Glass fiber
MEX	Material extrusion
*λ_E_*	Hybrid effect of the Young’s modulus
*ρ*	Density
*σ*_11_	Normal stress in x-direction
*σ*_22_	Normal stress in y-direction
SEM	Scanning electron microscope
*S_L_*	Longitudinal shear strength
*S_T_*	Transverse shear strength
*τ*_12_	Shear stress
*v*_12_	Poisson’s ratio
VAS	Volume average stiffness
*V*_*c*1_	Volume fraction of the first fiber composite system
*V*_*c*2_	Volume fraction of the second fiber composite system
*V* *_f_*	Fiber volume fraction
*v* *_f_*	Volume of the fibers in the specimen
*V* *_t_*	Volume of the specimen
*X_C_*	Longitudinal compressive strength
*X_T_*	Longitudinal tensile strength
*Y_C_*	Transverse compressive strength
*Y_T_*	Transverse tensile strength
